# Biosynthesis of Sciadonic Acid Derived from Gymnosperms with Anti‐Colitis Activity

**DOI:** 10.1002/advs.75502

**Published:** 2026-05-06

**Authors:** Yadi Gao, Wei Liu, Xiuping Liang, Jie Bai, Hong Chang, Wenchao Chen, Mohamed A. Farag, Jianbo Xiao, Huifeng Jiang, Jiasheng Wu, Lili Song

**Affiliations:** ^1^ State Key Laboratory for Development and Utilization of Forest Food Resources Zhejiang A&F University Hangzhou China; ^2^ Medical Engineering & Engineering Medicine Innovation Center, Hangzhou International Innovation Institute Beihang University Hangzhou China; ^3^ Tianjin Institute of Industrial Biotechnology Chinese Academy of Sciences Tianjin China; ^4^ University of Chinese Academy of Sciences Beijing China; ^5^ State Key Laboratory of Quality Research in Chinese Medicine, Institute of Chinese Medical Sciences University of Macau Macao China; ^6^ Pharmacognosy Department, College of Pharmacy Cairo University Cairo Egypt; ^7^ Department of Analytical Chemistry and Food Science, Faculty of Food Science and Technology University of Vigo Ourense Spain; ^8^ College of Biotechnology Tianjin University of Science & Technology Tianjin China

**Keywords:** Δ5 desaturase, colitis, metabolic engineering, sciadonic acid, *Yarrowia lipolytica*

## Abstract

Sciadonic acid (SCA), Δ5‐unsaturated fatty acid with anti‐inflammatory and lipid‐regulatory properties, is predominantly derived from seeds of gymnosperms. This study established a systematic engineered *Yarrowia lipolytica* platform for efficient SCA production and assessing the anti‐colitis efficacy of SCA as a novel therapeutic lipid. First, production of eicosadienoic acid (EDA) was biosynthesized by iterative expression of endogenous polyunsaturated fatty acid desaturase and Δ9 elongase. Different Δ5 desaturases were screened to identify high‐activity enzymes capable of catalyzing EDA‐CoA substrates, bypassing the rate‐limiting step of phosphatidylcholine (PC)‐bound substrate catalysis. Further, combined with the deletion of the β‐oxidation pathway, enhancement of triglyceride assembly and malonyl‐CoA supply, the resulting strain overproduced 195.5 mg/L SCA in shake flasks. Under optimized bioreactor fermentation, SCA yield reached 1.2 g/L with glucose, representing the high level of SCA production reported in *Y. lipolytica*. Finally, SCA significantly ameliorated dextran sulfate sodium salt (DSS) ‐induced colitis in mice, demonstrating dose‐dependent improvements in disease activity index, colon histopathology, and pro‐inflammatory cytokine suppression. Overall, this work establishes an efficient microbial production platform for SCA and provides in vivo evidence supporting the anti‐colitis potential of this bioactive lipid.

## Graphical abstract

This study establishes an efficient *Yarrowia lipolytica* platform for unusual sciadonic acid (SCA) production and assess the anti‐colitis efficacy in mice of SCA, supporting its potential as a novel therapeutic lipid.

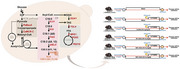

## 1 Introduction

Polyunsaturated fatty acids (PUFAs), renowned for their anti‐inflammatory, neuroprotective, and cardiovascular modulatory bioactivities, have emerged as pivotal functional molecules in nutraceutical and biomedical fields [1, 2, 3]. A novel subclass of PUFAs, Δ5‐Non‐methylene‐interrupted polyunsaturated fatty acids (NMIPUFAs), has recently sparked interest due to their structural uniqueness and therapeutic potential, resembling fish oil [4, 5]. Sciadonic acid (cis‐5, 11, 14‐eicosatrienoate acid, C20:3 Δ5, 11, 14, SCA) is an unconventional omega‐6 Δ5‐NMIPUFA enriched in the seed oil of gymnosperm plants [6]. It has demonstrated anti‐inflammatory properties [7, 8], triglyceride‐lowering effects in hyperlipidemic models [9], and to modulate gut microbiota composition [10]. Traditionally extracted from *Torreya grandis* seeds, SCA production faces challenges such as labor‐intensive purification and unstable yields [6, 11, 12] . These limitations underscore the urgency to develop sustainable SCA, given its promising anti‐inflammatory effects and potential as a high‐value bioactive lipid.

The biosynthesis of SCA involves coordinated different desaturation and elongation steps in high plants [6]. The pathway is initiated with oleic acid (C18:1 Δ9, OA), which undergoes Δ12 desaturation to form linoleic acid (C18:2 Δ9,12, LA). Subsequent elongation conversion of LA by Δ9 elongase forms eicosadienoic acid (C20:2 Δ11, 14, EDA), followed by Δ5 desaturase to generate SCA [6, 13, 14]. Metabolic engineering of microbial hosts offers a sustainable platform for high‐value natural product biosynthesis, such as terpenes, flavonoids, and fatty acids [15, 16, 17]. Engineered microbial chassis, particularly *Yarrowia lipolytica*, offer a promising platform by circumventing these limitations and enabling the high‐yield and sustainable production of PUFAs [18], such as docosahexaenoic acid (DHA) [19], eicosapentaenoic acid (EPA) [20], ‐γlinolenic acid (GLA) [21], and nervonic acid (NA) [22]. *Y. lipolytica* possesses inherent advantages for PUFA biosynthesis, such as a complete fatty acid elongation system and high oleic acid flux [23]. Its fatty acid synthase (FAS) complex produces acyl‐CoA precursors, which serve as substrates for elongase and desaturases. Xue et al. [24] achieved 56.6% EPA of total fatty acids (TFAs) via coordinated pathway engineering, while Sun et al. [21] produced 71.6 mg/L GLA by expressing *Mortierella alpina* Δ6 desaturase under low‐temperature fermentation. Wang et al. [25] further demonstrated the production of ω6‐PUFA yielding 386 mg/L GLA, 832 mg/L dihomo‐γ‐linolenic acid (DGLA), and 192 mg/L arachidonic acid (ARA) via combinatorial desaturase expression and precursor optimization. Given *Y. lipolytica's* proven capacity to express diverse desaturases and assemble complex lipids, we propose it as a potential host for SCA biosynthesis.

In this study, we first constructed the biosynthetic pathways of SCA in *Y. lipolytica* by multi‐modular metabolic engineering strategies and enzyme screening. In parallel, to evaluate the biological relevance of SCA, we assessed the anti‐colitis activity of an SCA‐enriched preparation in a DSS‐induced mouse model. This combined strategy enabled us to address both the feasibility of microbial SCA production and the in vivo therapeutic potential of SCA.

## 2 Materials and Methods

### 2.1 Genes and Strains

All metabolically modified strains of *Y. lipolytica* were derived from the W29 strain, which harbored the *Cas9* gene integrated into the KU70 locus. All heterologous genes were codon‐optimized for *Y. lipolytica* and synthesized by GenScript (Nanjing, China). Integration sites, pTEFin and pGPD promoters, tLIP2 and tPEX20 terminators were amplified from *Y. lipolytica* genomic DNA. All primers and plasmids were listed in Tables S1 and S2, and strains were listed in Table S3.

### 2.2 Cultivation and Medium

*Escherichia coli* strains were grown in Lysogeny Broth (LB) medium at 37 °C with agitation at 220 rpm. *Y. lipolytica* strains were incubated using YPD medium (10 g/L yeast extract, 20 g/L peptone, 20 g/L glucose) at 30 °C with agitation at 220 rpm. For selection of transformants, YPD medium was supplemented with nourseothricin (400 mg/L) and hygromycin B (250 mg/L). Shake flask batches were conducted in 250 mL containers with 30 mL YPD medium (50g/L glucose). All fermentation results represent mean ± standard deviation from three biologically independent replicates.

### 2.3 Genetic Engineering

Gene knockout and integration were performed by using a CRISPR/Cas9 system with gRNA plasmids carrying specific 20‐bp targeting sequences designed for optimal expression efficiency according to Holkenbrink et al. [26]. All target sequences were designed by the CHOPCHOP (http:// chopchop.cbu.uib.no). Donor DNAs for gene deletion and integration were assembled by Gibson assembly PCR: knockout donors featured about 1 kb upstream and downstream homologous arms, while integration donors contained assembled expression cassettes flanked by homologous arms. Transformation of *Y. lipolytica* cells was performed using lithium acetate method following Chen et al. [27].

### 2.4 Lipid Extract and Fatty Acid Analysis

Culture cells were pelleted by centrifugation and subjected to freeze‐drying under vacuum (0.1 mbar, −80 °C) to obtain cell biomass. Total lipids were extracted by the Folch method, followed by transesterification using 1 mL heptane to generate fatty acid methyl esters (FAMEs), as optimized by Zhang et al. [28]. Gas Chromatography (GC) analysis of fatty acid methyl esters was performed using flame ionization detector (FID) equipped with a capillary column (DB‐wax UI, 30 m × 0.25 µm × 0.25 mm, Agilent). A sample of 1 µL was injected with the injector at 250 °C, column temperature was raised from 100 °C to 240 °C at 15 °C min^−1^, and finally kept for 5 min. The FID detector temperature was set at 250 °C. Fatty acid standards for methyl palmitate (C16:0), methyl stearate (C18:0), methyl oleate (C18:1), methyl linoleate (C18:2), eicosadienoic acid methyl ester (C20:2 Δ11,14), and methyl eicosatrienoic acid methyl ester (C20:3 Δ5,11,14) were purchased from Sigma Aldrich (Shanghai, China) and used as standards to identify fatty‐acid peaks. Total lipid content of samples with different fatty‐acid methyl esters was calculated using fatty‐acid standards.

### 2.5 Determination of Distribution of Fatty Acids in TAG and PC

The fatty acid distribution of TAG and PC was analyzed as previously described [29]. Briefly, total lipids were dissolved in methanol and separated by thin‐layer chromatography (TLC) on Partisil K6 Silica gel 60 plates (250 µm thickness, 20 × 20 cm; Merck) using a developing solvent system of chloroform:methanol:water (62:25:4, v/v). Lipid bands were visualized by brief exposure to iodine vapor. The bands corresponding to TAG and PC were then scraped and converted to methyl esters by incubation with 2% (v/v) H_2_SO_4_ in methanol at 85°C for 3 h. The resulting FAMEs were analyzed by GC as described above.

### 2.6 Separation and Purification of SCA

Freshly pressed *T. grandis* seed oil was subjected to transesterification. The oil was combined with 100% methanol and potassium hydroxide (4:1 methanol‐to‐oil ratio with 1% KOH w/w) and reacted in a stirrer at 40°C for 40 min. The mixture was transferred to a separatory funnel and allowed to phase‐separation for 30 min before discarding the glycerol layer. The FAME phase was sequentially washed with saturated sodium chloride solution and deionized water. Anhydrous magnesium sulfate was added to remove residual water, followed by filtration. Multi‐stage molecular distillation was performed using a short‐path distillation system (KDL2‐UIC Short Path Distillation, Germany) under optimized conditions: evaporator temperature of 100°C, wiper speed of 200 rpm, feed rate of 3 s/d, condenser temperature at 20°C, and vacuum pressure of 1.0 × 10^−3^ MPa. After 4 sequential distillation cycles, the distillate fractions were collected. The percentage of SCA methyl ester in the final fraction was quantified by GC‐FID as described in 2.4.

### 2.7 Animal Studies

Male C57BL/6 mice (8 weeks old, 18 – 22 g) were group‐housed under controlled conditions (25 ± 1°C, 12‐h light/12‐h dark cycle) with unrestricted access to standard diet and tap water (or specified drinking solution). The mice were housed at the laboratory of Traditional Chinese medicine, Zhejiang Agriculture and Forestry University (Hangzhou, Zhejiang, China). After 7‐day acclimatization, colitis was induced by administering 2.7% DSS in water. For the animal study, SCA was administered orally as an SCA‐enriched oil fraction obtained from *T. grandis* seed oil after molecular distillation, without an additional external vehicle. Different SCA percentages in the administered preparation were achieved by controlling the molecular distillation process. Experimental groups (n = 10) were conducted to include: high‐dose SCA (50% SCA, 10 mL/kg/day) and medium‐dose SCA (30% SCA, 10 mL/kg/day), low‐dose SCA (9% SCA, 10 mL/kg/day), positive control (5‐Amino salicylic acid, 5‐ASA, 100 mg/kg/day). Treatments were co‐administered with DSS throughout the induction period. Animal experiments were approved by the Animal Welfare and Ethics Committee of the Zhejiang Academy of Agricultural Sciences (Ethics protocol no. 1935) and conducted in compliance with Chinese guidelines for the care and use of laboratory animals (protocol number, ZAFUAC2022015) and Zhejiang Farm Animal Welfare Council principles.

During the experiment, the body weight and food intake of the mice were measured at regular intervals every day. The comprehensive weight loss rate of each group, the softness and hardness of the feces of mice, and the condition of anal bleeding were used as indices to evaluate the severity of enteritis. After the 3^rd^ week, all the experimental mice were sacrificed under anaesthesia, and cecal, colon tissues and serum samples of all the mice were collected, and stored in a refrigerator at ‐80°C for further assays. A preliminary acute safety evaluation was performed by orally administering the SCA‐enriched preparation for 14 days, followed by assessment of survival, body weight, and gross morphology of major organs.

### 2.8 Histological Examinations

Mice were terminally anesthetized with sodium pentobarbital (50 mg/kg i.p.) and transcardially perfused with 4% paraformaldehyde in phosphate buffer (pH 7.2). Colon was removed, longitudinally incised, cleared of contents, rolled using a wooden stick technique [30], and immersion‐fixed. Tissues were routinely processed using ethanol dehydration, paraffin embedding, and sectioned at 4 µm for standard hematoxylin and eosin (H&E) histology.

### 2.9 Assay of Inflammatory Mediators

Serum levels of TNF‐α, IL‐6, IL‐1β, and MCP‐1 were quantified using commercial enzyme‐linked immunosorbent assay (ELISA) kits (Thermo Fisher Scientific, Cat#88‐7324, 88–7064, 88–7013, Waltham, Massachusetts, USA; Servicebio, GEM0017, Wuhan, China). The assays were performed according to the manufacturer's protocols with duplicate measurements. Absorbance was read at 450 nm using a microplate reader, and cytokine concentrations were calculated against standard curves.

### 2.10 Fed‐Batch Fermentations

Bioreactor fermentation was carried out in a 7.5‐L bioreactor (Shanghai Baoxing Bio‐Engineering Equipment, Shanghai, China). The fermentative medium contained 100 g/L glucose, 5 g/L (NH4)_2_SO_4_, 3 g/L KH_2_PO_4_, 0.5 g/L MgSO_4_·7H_2_O, 2 mL/L trace metals solution and 1 mL/L vitamins solution. The pH was automatically maintained at 5.0 by adding 4 M KOH, and dissolved oxygen (DO) was controlled at 30% atmospheric saturation through automatic adjustment of the agitation speed (200 to 800 rpm). During the process of fermentation, 2 mL of cell samples were collected every 12 h. The supernatant was used for glucose concentration determination by an SBA‐40D Biosensor (Shandong Academy of Sciences, Jinan, China), and the pelleted cells were washed twice and freeze‐dried to measure the dry we. Lipid content and SCA titer were extracted and quantified as described above.

## 3 Results

### 3.1 Construction of De Novo Precursor of SCA Biosynthetic Pathway

**FIGURE 1:**
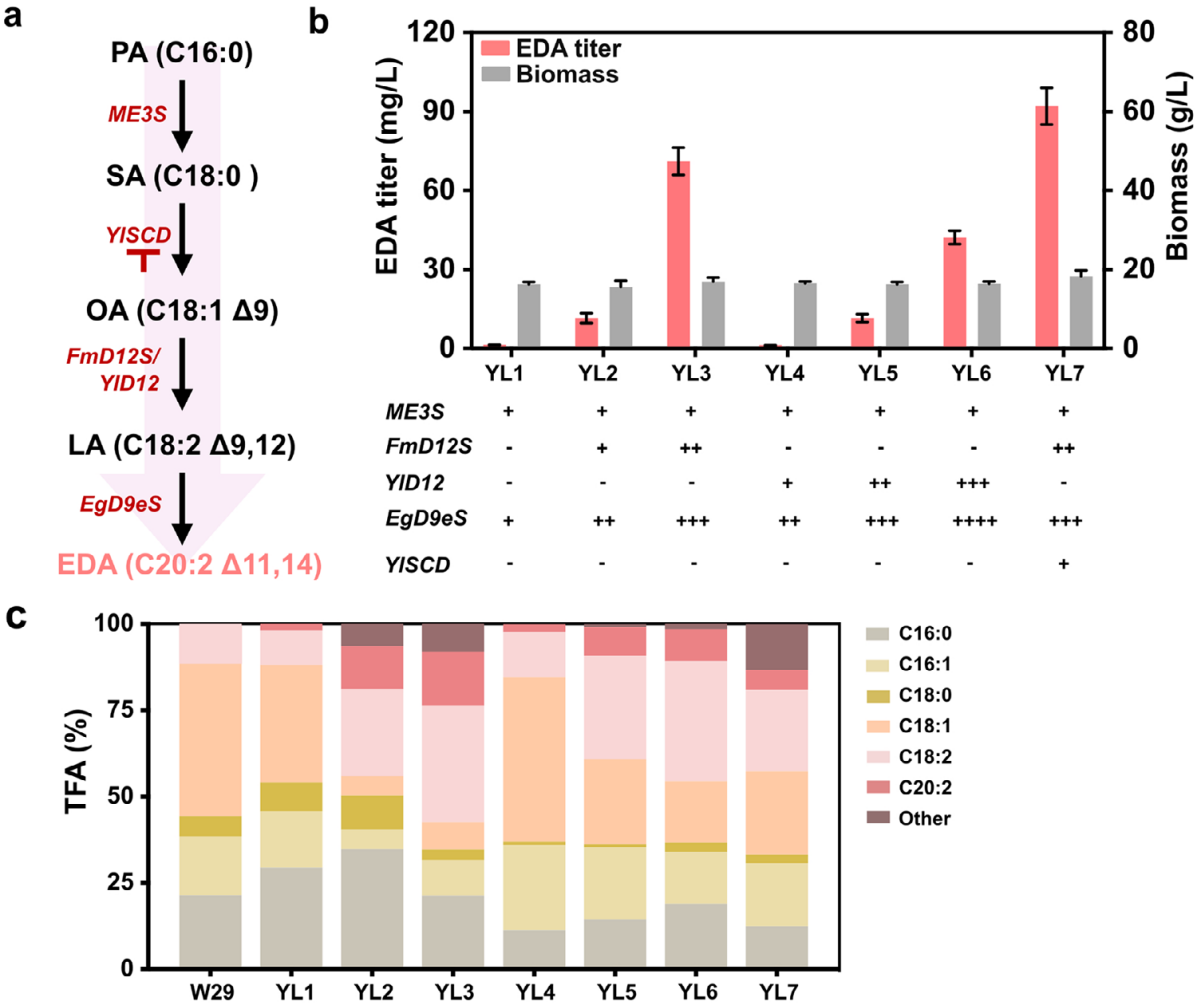
Construction of *de novo* precursor of SCA biosynthetic pathway in *Y. lipolytica*. (a) Schematic illustration of the upregulation of polyunsaturated fatty acid biosynthesis. PA, palmitic acid; SA, stearic acid; OA, oleic acid; LA, linoleic acid; EDA, eicosadienoic acid. *ME3S*, C16/19 elongase from *Mortierella alpina*; *YlSCD*, stearoyl‐CoA desaturase from *Y. lipolytica*; *FmD12S*, fatty acid desaturase 2 from *Fusarium monoliforme*; *YlD12*, *fatty acid desaturase 2* from *Y. lipolytica*; *EgD9eS*, Δ9 elongase from *Euglena gracilis*. (b, c) Effect of the overexpressed genes of the polyunsaturated fatty acid biosynthesis pathway on the EDA titer and percentage of total fatty acids. All data represent the means of n = 3 biologically independent samples and error bars show standard deviations.

In order to build a *Y. lipolytica* chassis with the capacity to produce high levels of SCA, engineered PUFA biosynthesis pathway was constructed to enhance its precursor EDA production (Figure 1a). Specifically, codon‐optimized sequences of C16/18 elongase coding gene *ME3S* and Δ9 elongase gene *EgD9eS* were integrated into the genome of wild type W29 strain, resulting in strain YL1. GC‐MS analysis revealed that YL1 yielded 1.49 mg/L EDA (1.59% of TFAs) after 3‐day fermentation (Figure 1b), confirming the functional expression of the heterologous enzymes.

The metabolic flux towards the downstream pathway was enhanced by screening and multicopy integration of key genes. Different microbial Δ12 desaturases were introduced into the YL1 strain controlled by *TEFIN* promoter accompanied by overexpressed *EgD9eS*. Dual‐copy overexpression of *FmD12S* and *EgD9eS* elevated EDA to 15.55% of TFAs (71.22 mg/L), a 7.9‐fold increase over single‐copy expression, while generating α‐linolenic acid (ALA) as a byproduct due to its dual desaturase activity [31]. Increasing the copy number of endogenous *YlD12* enhanced EDA production by 9.5‐fold, while significantly reducing byproduct formation. Nevertheless, YL6 (3 copies) exhibited no significant EDA increase (9.29% TFAs, 42.33 mg/L) compared to YL3, indicating limited benefits from high‐copy endogenous *YlD12*. Moreover, the minimal alteration in biomass suggested that the improved copy number of *YlD12* and *EgD9eS* did not impose an additional metabolic load on the strains (Figure 1b). On the other hand, the ratio of unsaturated to saturated fatty acid was shown to increase in the *YlSCD*‐overexpressed strain (Figure 1C). Although the EDA percentage of TFA was decreased, lipid content increased by 1.7 times, and the yield of EDA was increased to 92.09 mg/L, which was 1.3 times higher than that in YL3 (Figure 1b, c).

### 3.2 Unlocking the Rate‐Limiting Desaturase Bottleneck in SCA Biosynthesis

**FIGURE 2:**
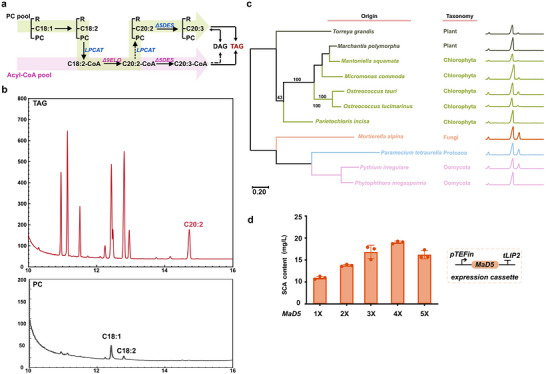
Unlocking the rate‐limiting desaturase bottleneck in SCA biosynthesis. (a) Schematic illustration of the elongation and desaturation of SCA biosynthesis from C18 fatty acid in *Y. lipolytica*. PC, phosphatidylcholine; DAG, diacylglycerol; TAG, triacylglycerol. *LPCAT, lysophosphatidylcholine acyltransferase*; *Δ9ELO*, *Δ9 elongase*; *Δ5DES*, *Δ5 desaturase*. (b) GC trace of FAME derived from the TAG fraction of *Y. lipolytica* EDA chasis strain YL7. (c) Phylogenetic analysis and the functions of Δ5 desaturases for SCA biosynthesis. (d) Effect of optimizing *MaD5* copy numbers on SCA production. All data represent the means of n = 3 biologically independent samples and error bars represent standard deviations.

A fundamental distinction between PUFA desaturases from higher plants and microbes lies in their substrate preference, with plant enzymes typically acting on phosphatidylcholine (PC)‐bound fatty acid CoA, whereas their microbial counterparts often utilize acyl‐CoA substrates (Figure 2a). To determine whether *Y. lipolytica* chassis was capable of producing SCA in PC pool, total lipids were then extracted from the YL7 cells and were fractionated into PC or TAG classes by TLC (Figure 2b). For total lipids, 21% of EDA had been elongated with no enrichment in the PC fraction compared with the total lipid extract. The absence of EDA in the PC fraction indicated that the plant‐derived Δ5 desaturase was unable to effectively utilize the PC‐bound substrate in *Y. lipolytica*, suggesting the necessity to screen for more efficient Δ5 desaturases capable of directly converting C20:2‐CoA. Therefore, the identification and characterization of specific Δ5 desaturases functioning efficiently as acyl‐CoA desaturases remain a critical bottleneck in constructing high‐yielding SCA cell factories.

Overexpression of *TgDES1* in the EDA chassis resulted in a desaturase conversion rate of only 10.79% (Figure 2c). Considering the substrate dichotomy of high plant desaturase TgDES1, heterologous expression of lysophosphatidylcholine acyltransferase (LPCAT) from different species failed to increase desaturase rate (Figure S1), suggesting intrinsic limitations in phospholipid substrate channeling [14]. In contrast, substrate dichotomy necessitates systematic screening of phylogenetically diverse desaturases to identify enzymes capable of circumventing substrate channeling bottlenecks for efficient SCA production (Figure 2a; Table S4). Synthases from different evolutionary branches were selected to evaluate their functions and efficiency by chromosomally integrating them into strain YL7. MaD5 (from *M. alpina*) and Ptet1D5D (from *Paramecium tetraurelia*) exhibited 30.41% and 27.33% Δ5 desaturation efficiency, which were 2.8‐ and 2.5‐fold higher than TgDES1, respectively.(Figure 2c) [32, 33, 34]. Inspired by the dose‐responsive flux regulation in yeast fatty acids production [24], a strain with multi‐copy *MaD5* was engineered. It was then speculated that increasing the copy number of *MaD5* could shuffle more EDA‐CoA toward SCA biosynthesis. The yield of SCA in strain YL25 expressing 4 genomic copies of *MaD5* reached 18.89 mg/L (18.61% conversion rate), demonstrating the superior substrate specificity for EDA (Figure 2d).

### 3.3 Redirecting Lipid Flux in the SCA Biosynthetic Pathway

**FIGURE 3:**
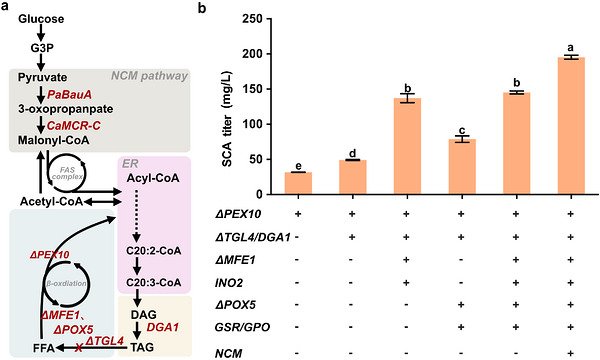
Redirecting lipid flux in the SCA biosynthetic pathway (a) The assemble and degradation of SCA‐enriched TAG in ER catalyzed by elongase and desaturase from glucose. G3P, glyceraldehyde‐3‐phosphate; DAG, diacylglycerol; TAG, triacylglycerol; FFA, free fatty acids; NCM pathway, Non‐carboxylated Malonyl‐CoA pathway; ER, endoplasmic reticulum. *PaBauA, β‐alanine‐pyruvate transaminase*; *CaMCR‐C*, *C‐terminal fragment of malonyl‐CoA reductase*; *DGA1*, *diacylglycerol acyltransferase 1*; *TGL4, triglyceride lipase 4*; *PEX10*, peroxisomal membrane protein 10; *MFE1*, *multifunctional β‐oxidation hydratase/dehydrogenase 1*; *INO2*, inositol‐1‐phosphate synthase 2, which positively regulated ER size; *POX5*, *peroxisomal acyl‐CoA oxidase 5*; *GSR*, *glutathione disulfide reductase*; *GPO*, *glutathione* peroxidase. Dashed arrows indicate multi‐step pathways. (b) SCA titer of *Y. lipolytica* engineered strains. All data represent the means of n = 3 biologically independent samples and error bars show the standard deviations.

**FIGURE 4:**
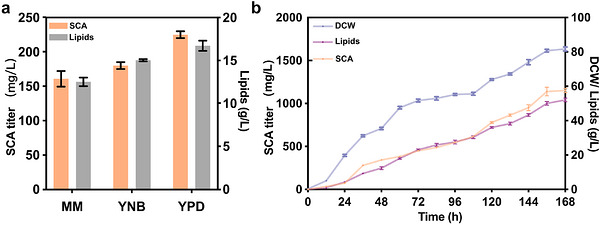
Fed‐batch fermentation of the engineering strain in a 5‐L bioreactor. (a) Shake flask fermentation of engineered strain YL32 in different media. (b) SCA production by the engineered strain YL32 in a 5‐L bioreactor using MM medium. All data represent the means of n = 3 biologically independent samples and error bars show the standard deviations.

To further redirect more metabolic flux to SCA biosynthesis, related precursor supply and lipid assembly were screened by “push‐pull” strategy. In *Y. lipolytica*, peroxisomes oxidize long‐chain fatty acids via β‐oxidation [36]. As shown in Figure 3a, b, targeted disruption of *PEX10*, a key peroxisome biogenesis gene responsible for peroxisomal matrix protein and β‐oxidation, increased the SCA titer in YL27 by 2.3‐fold (to 32.35 mg/L) compared with strain YL11 (Figure 3b). To improve the lipid content for further accumulation of SCA, the diacylglycerol acyltransferase gene (*DGA1*) was overexpressed and TAG lipase encoding gene (*TGL4*) was deleted to enhance the assemble TAG levels, and to block TAG degradation. As shown in Figure 4b, the content of SCA in YL28 reached to 47.25 mg/L (Figure 3b).

Previous studies have shown that overexpression of *INO2*/*INO4* transcription factor activated endoplasmic reticulum (ER) structural alteration, alongside improved production of terpenoids and unsaturated fatty acids in yeast [22, 37]. In addition, the susceptibility of PUFA‐rich lipids to free radical‐mediated peroxidation disrupts cellular homeostasis, and ultimately impairs metabolic efficiency and cell viability [38, 39]. By upregulating oxidative stress defense pathways including glutathione disulfide reductase gene (*GSR*) and glutathione peroxidase (*GPO*), the *Y. lipolytica* cell morphology was improved [40]. The multifunctional β‐oxidation hydratase/dehydrogenase (*MFE1*) was involved in the β‐oxidation and degradation pathway of fatty acid [41]. Thus, overexpression of *YlINO2* with the deletion of *MFE1*, YL29 resulted in a 1.78‐fold increase in SCA titer. With the overexpression of *YlGPO* and *YlGSR* and deletion of *pox5*, the engineered strain had a similar proportion of SCA, but the oil content was significantly increased to 1.6 times (Figure 3b; Figure S2). Malonyl‐CoA acts as an indispensable substrate for the biosynthesis of PUFAs, providing carbon units and acyl groups essential for chain elongation [42]. The NCM (non‐conventional malonyl‐CoA) pathway employs a synthetic metabolic route that converts pyruvate into malonyl‐CoA via 3‐oxopropanoate as an intermediate, bypassing the conventional acetyl‐CoA‐dependent pathway [43]. The result showed that the NCM pathway can elevate malonyl‐CoA pool availability, yielding a 1.4‐fold increase in SCA titer (Figure 3b).

### 3.4 Fed‐Batch Fermentation of the Engineering Strain in 5‐L Bioreactor

Medium optimization is critical for enhancing heterologous chemical production in *Y. lipolytica* [44]. In this study, three culture media (YPD, YNB, and MM) were systematically assessed for the fermentation performance of engineered strain YL32 in shake flasks with 50 g/L glucose. As shown in Figure 4a, YPD medium yielded the highest SCA titer (225 mg/L, 2.69% of TFAs) and lipid accumulation (8.36 g/L), while MM medium exhibited moderate growth but maintained comparable SCA percentage (2.57%) (Figure 4a). Given the trade‐offs between cost‐effectiveness and process scalability, MM medium was selected for subsequent fed‐batch fermentation to balance economic viability and bioreactor operation.

The initial glucose concentration was set at 100g/L, and an 800 g/L glucose and ammonium sulfate solution was supplemented continuously from 24 to 72 h. The fermentation process was dynamically controlled by adjusting pH to 5.5 and the DO level to 30% of atmospheric saturation. Upon glucose depletion, the rebound in DO serve as a trigger to initiate nutrient supplementation, with residual glucose concentration controlled at 10 g/L to sustain cellular metabolism and growth. As shown in Figure 4b, maximal total lipid content reached 81.67 g/L of DCW at about 168 h, representing a 2‐fold increase compared with the shake flask results. The final strain accumulated 52.00 g/L of lipids, including 1.2 g/L SCA, representing 7.4 folds higher than in shake flasks (Figure 4b).

### 3.5 SCA Alleviates DSS‐Induced Ulcerative Colitis in Mice through Anti‐Inflammatory Mechanisms

Considering SCA anti‐inflammatory potential activity in general inflammatory models [45, 46], its efficacy in colitis‐specific inflammation remains unexplored. To obtain preliminary information regarding in vivo safety, we performed a short‐term acute safety evaluation of the SCA‐enriched preparation. No mortality was observed during the test period, body weight increased normally, and gross examination of major organs did not reveal obvious abnormalities (Table S5). These data support a favorable preliminary acute safety profile of the tested preparation under the evaluated conditions. To better link exposure to pharmacological efficacy, we further analyzed the pharmacokinetic and tissue distribution profile of SCA after oral administration (Figure S3; Table S6). SCA was rapidly absorbed into systemic circulation, reaching a peak plasma concentration (C_max_ = 144.5 ng/mL) at 1 h (T_max_). However, it was also rapidly cleared from plasma, with a short half‐life (t_1/2_ = 0.94 h) and a high clearance rate (Cl = 40637.25 mL/h/kg), indicating extensive first‐pass metabolism and systemic elimination. In contrast, SCA accumulated and was retained in the target colon tissue. The colon T_max_ was delayed to 6 h, and the terminal half‐life in the colon was 10 times (t_1/2_ = 13.48 h) longer than in plasma. This distribution pattern provides a plausible exposure basis for the observed anti‐colitis activity of SCA in the intestine.

**FIGURE 5:**
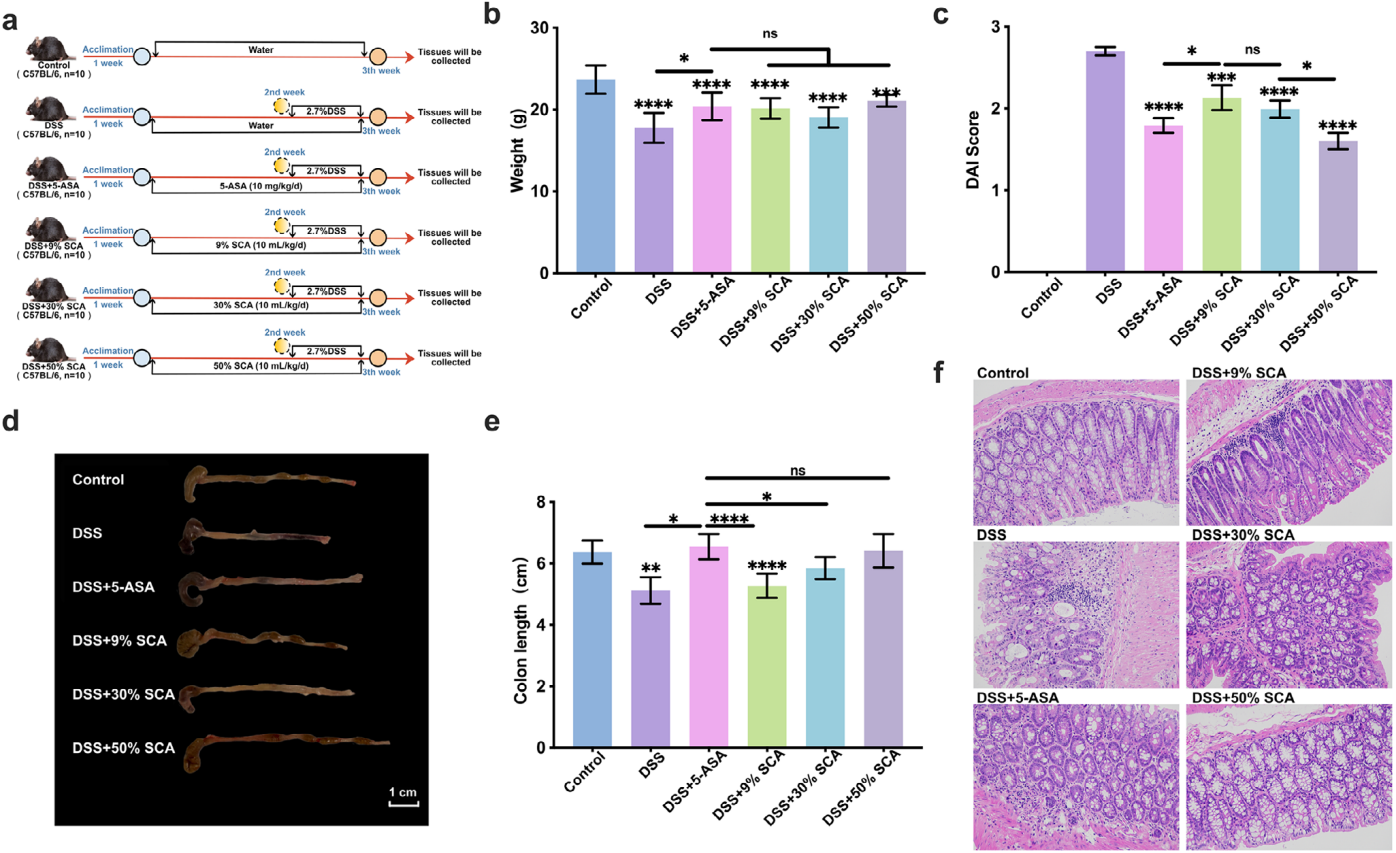
SCA alleviates symptoms of DSS‐induced ulcerative colitis in mice. (a) Schematic diagram of the experiment design. DSS, dextran sulfate, Positive group: 5‐ASA. (b) Body weight changes in mice across different treatment groups. (c) Disease activity index (DAI) score. (d) Macroscopic view of mouse colon length; (e) Statistical analysis of colon length. (f) Hematoxylin and eosin (H&E)‐stained colon sections of DSS and different SCA treatments in the colon (200 ×). All data represent the means of n = 10 biologically independent samples. Data are presented as mean ± SD; *P < 0.05 versus Control group; **P < 0.01 versus Control group, ***P < 0.001 versus Control group, ****P < 0.0001 versus Control group (one‐way ANOVA with Tukey's test).

Here, we pioneer the evaluation of the extracted SCA in a DSS‐induced ulcerative colitis (UC) mouse model. C57BL mice (n = 10/group) were received 2.7% DSS with/without 5‐ASA (positive group, 100 mg/kg/d) or SCA (9%, 30% or 50%, 10 mL/kg/d) for 7 days (Figure 5a). DAI score, body weight and colon length collectively represent the severity of colitis in animal models directly. The body weight and DAI scores of each mice group after 7 days of treatment are presented in Figure 5b–e. DSS led to a significant decrease in body weight of the model mice group, with severe hematochezia and diarrhea. There were remarkable differences in mice's weight (*p* < 0.0001) between the model and the control groups. However, the symptoms of diarrhea, weight loss, and fecal occidental blood were improved in all the low‐, medium‐ and high‐dose SCA groups. The overall effects of SCA on colon condition were also observed (Figure 5d). While the colon length of the model group was significantly shorter than that of the normal group (*p* < 0.01), the colon condition of the 5‐ASA and SCA groups was improved, with significantly increased colon length and less edema. With the increase of SCA concentration, both physical condition and colon length improved significantly and were found comparable to the positive drug 5‐ASA group.

The eroded intestinal epithelial cells induced by DSS administration could increase the colonic mucosal permeability. Compared with the normal group, the pathological section of colon tissues of mice in the model group showed intestinal wall edema and thickening, with a considerable number of inflammatory cells infiltration, severely deformed crypt structure, and destroyed or even disappeared goblet cells (Figure 5f). Notably, SCA significantly ameliorated DSS‐induced histopathological damage in colonic tissues compared to the model group, as evidenced by reduced crypt distortion and preserved goblet cell density (Figure 5f). TNF‐α, IL‐6, IL‐1β, and MCP‐1 are key pro‐inflammatory cytokines that orchestrate immune responses by recruiting leukocytes and amplifying inflammatory cascades in many diseases [47]. In this study, TNF‐α, IL‐6, IL‐1β and MCP‐1 were decreased significantly in the 30% and 50% SCA with DSS‐induced group, while IL‐6 and IL‐1β showed no significant difference compared with the control group (Figure S4). Transcriptomic profiling of colonic tissues from high‐dose SCA‐treated versus DSS‐induced UC mice provides the first evidence of SCA‐mediated rescue via nutrient metabolic restoration. KEGG pathway enrichment analysis revealed upregulation of key processes involved in protein, fat, carbohydrate, and vitamin digestion and absorption, alongside pancreatic secretion in high‐dose SCA group (Figure S5). To further determine whether SCA contributed to intestinal barrier recovery, we examined the expression of the tight junction markers ZO‐1 and Occludin in colonic tissues via RT‐qPCR and Western blot. DSS treatment markedly reduced the expression of both markers at the mRNA and protein levels (*P* < 0.05). In contrast, high‐dose SCA treatment significantly restored ZO‐1 and Occludin expression, with effects comparable to those observed in the 5‐ASA group (*P* < 0.05, Figure S6a, b). Western blot results showed that compared with control group, the expression of tight junction proteins ZO‐1 and Occludin were decreased remarkably in model group (*P* < 0.05), and 5‐ASA, 50% SCA significantly increased ZO‐1 (*P* < 0.05) and Occludin expressions (*P* < 0.05). However, 30% SCA increased slightly the protein expressions of ZO‐1 (*P* > 0.05) and Occludin1 (*P* < 0.05) compared to DSS group (Figure S6c, d). These results indicate that SCA also promotes barrier repair in the injured colon by upregulating ZO‐1 and Occludin.

## 4 Discussion

SCA represents an immunomodulatory Δ5‐desaturated polymethylene‐interrupted fatty acid with significant therapeutic potential for inflammatory [7, 48]. However, its broader application is constrained by the limitations of traditional plant extraction methods, which are unsustainable, yield‐dependent on climatic factors, and difficult to standardize for industrial‐scale production [6, 14]. Here, we systematically engineered *Y. lipolytica* for *de novo* biosynthesis of SCA by functionally identifying biosynthetic enzymes and optimizing metabolic flux. Notably, the engineered strain in a 1 m^3^ produced fermenter an amount of SCA equivalent to the yield from *T. grandis* cultivated on approximately 120,000 m^2^ of land (details of calculations were added to the Text S1). These results highlighted a scalable and efficient route to achieve high‐purity SCA, and fermentation‐based process ensures a fresh, oxidatively stable supply, making it a more reliable “plant‐alternative” for nutraceutical development where dose consistency is paramount.

The functional characterization of key elongases and desaturases was critical for the efficient heterologous production of PUFA in microbial chassis [24]. The 9.5‐fold EDA yield increase in strain YL3 underscores the advantage of Δ12 desaturase (*YlD12*) and Δ9 elongase (*EgD9eS*) in this yeast, while the 1.7‐fold lipid content boost from *YlSCD* overexpression suggests improved membrane fluidity facilitated metabolic flux. In contrast, plant‐derived Δ5 desaturases showed low activity in yeast (Figure 2) [6, 14]. To overcome the Δ5‐desaturation bottleneck, we screened phylogenetically diverse desaturases and identified MaD5 from *M. alpina*, a highly efficient candidate (Figure 2C). The superior performance of MaD5 in *Y. lipolytica* may reflect improved compatibility with the yeast intracellular environment, including more efficient folding, membrane insertion, and coupling to the host endoplasmic reticulum‐associated lipid metabolic network [10, 49]. In addition, the efficiency of Δ5‐desaturation may depend on substrate accessibility within the acyl‐CoA pool and on host‐specific acyl flux distribution [50, 51]. These possibilities remain speculative at present, and further biochemical studies will be required to clarify the relative contributions of enzyme structure, localization, and metabolic coupling [52, 53, 54]. However, this should not be interpreted as evidence of a universal phylogenetic rule. Phylogenetic origin may help guide candidate selection, but empirical screening remains essential for identifying the best‐performing desaturase in a specific metabolic chassis.

A “push‐pull” strategy synergistically redirected metabolic flux significantly increasing in natural products titer [55]. Disrupting β‐oxidation (*pox5*, *PEX10* and *MFE1*) increased SCA by 2.3‐fold by reducing precursor competition, while enhancing TAG storage by overexpressed *DGA1* and *TGL4* knockout created a sink for lipid accumulation, demonstrating the need for coordinated pathway engineering. Previous studies have established that the elongation and desaturation of PUFAs mainly occur on the ER and overexpression of *INO2* can significantly expand ER size and enhance cellular stress response in yeast [22, 37]. PUFAs are particularly susceptible to oxidation due to the presence of carbon‑carbon double bonds in their structure [40]. Therefore, the synergy between *YlINO2* overexpression and redox defense genes (*YlGPO* and *YlGSR*) underscores that redox homeostasis is integral to sustaining production of SCA. The engineered strain with genomic engineering demonstrated stable and high titer over the short term (Figure 4), supporting its potential for pilot‑scale expansion. Further long‑term validation will ultimately confirm its suitability for industrial‑scale fermentation. Beyond SCA production, the engineering framework established here may be extendable to other Δ5‐unsaturated fatty acids. The combination of precursor supply enhancement, host‐compatible desaturase selection, acyl flux redistribution, and storage‐lipid reinforcement provides a modular architecture.

UC is a chronic condition characterized by unpredictable cycles of disease activity and remission [56]. Long‐term use of chemical drugs can lead to gastrointestinal discomfort and impairments in liver and kidney function. Previous studies have suggested that SCA can modulate inflammatory signaling pathways by incorporating into cell membrane phospholipids [9, 45]. For instance, following its incorporation into host epithelial phospholipids, SCA reduces PGE2 synthesis by downregulating COX‐2 expression or competitively inhibiting the ARA metabolic pathway. Concurrently, it suppresses MAPK phosphorylation and NF‐κB translocation, thereby blocking the inflammatory cascade [8, 57]. Similarly, another Δ5 PUFA juniperonic acid inhibits the release of inflammatory mediators such as NO, IL‐6, and TNF‐α, and downregulates MAPK pathway activity via an analogous membrane incorporation mechanism [58]. The inhibitory effect of SCA on pro‐inflammatory factors observed in this study is highly consistent with the above mechanisms, suggesting that SCA may also influence MAPK/NF‐κB signal transduction by modulating the composition of cell membrane phospholipids. Furthermore, the activation of nutritional metabolic pathways of transcriptomic analysis suggests that SCA may indirectly inhibit inflammatory responses by improving intestinal barrier function and energy metabolism (Figure S2). Moreover, the intestinal barrier is the first line of defense against external pathogenic substances such as bacteria and viruses [59]. Tight Junction Proteins (including ZO‐1 and Occludin) are important structures that maintain the tight junctions between intestinal epithelial cells and ensure the integrity of the intestinal barrier [60]. RT‐qPCR and Western blot detections showed that 50% SCA could enhance the expression of tight junction proteins ZO‐1 and Occludin in the colon (Figure S6).

## 5 Conclusion

In conclusion, this study combined precursor optimization, Δ5‐desaturase screen and pathway redirection to obtain gram‐scale SCA biosynthesis (1.2 g/L), representing the first reported high‐level Δ5‐UPIFA in *Y. lipolytica*. Compared with plant chassis, it shows higher production capacity and efficiency in the production of SCA. High‐dose SCA exhibits significant anti‐colitis efficacy in a mouse model. These findings collectively validate oleaginous yeasts as sustainable and cost‐effective platforms for production of bioactive lipids and position SCA as a promising therapeutic candidate for further development. Differences in the surrounding lipid matrix between plant‐derived and future yeast‐derived preparations may influence formulation properties and should be addressed in future studies.

## Funding

This work was financially supported by the National Natural Science Foundation of China (U23A20219), National Key Research and Development Program of China (2022YFD2200603), Zhejiang Provincial Academy Cooperative Forestry Science and Technology Project (2025SY02) and the 111 Project (D18008).

## Conflicts of Interest

The authors declare no conflict of interest.

## Supporting information




**Supporting File 1**: advs75502‐sup‐0001‐SuppMat.docx.

**Supporting File 2**: advs75502‐sup‐0002‐FigureS1.docx.

**Supporting File 3**: advs75502‐sup‐0003‐TableS1.xlsx.

## Data Availability

The data that supports the findings of this study are available in the supplementary material of this article.
