# Supplementary material for: Biosynthesis of Sciadonic Acid Derived from Gymnosperms with Anti‐Colitis Activity
Source: Adv Sci (Weinh). 2026 May 6;13(42):e75502. doi: 10.1002/advs.75502 (PMC13335445; doi:10.1002/advs.75502)
Supplement: Supplementary file 1 — Supporting File 1: advs75502‐sup‐0001‐SuppMat.docx. [file ADVS-13-e75502-s002.docx]

Supplementary text1

Currently, the planting and nut harvest cycle of *T. grandis* is 10 years and the nut yield is about 750 kg/ha (~34% oil yield). The proportion of SCA in the total fatty acid is 9.13~12.96% and the yield is 2.5 kg/ha/yr. In our system, the fermentation cycle is 14 days and the productivity of SCA is 1.2 g/L in 5 L bioreactor. The output of fermenter is approximately equal to 1.2 * 2 * 12 = 28.8 kg/yr.
