# Supplementary material for: Biosynthesis of Sciadonic Acid Derived from Gymnosperms with Anti‐Colitis Activity
Source: Adv Sci (Weinh). 2026 May 6;13(42):e75502. doi: 10.1002/advs.75502 (PMC13335445; doi:10.1002/advs.75502)
Supplement: Supplementary file 2 — Supporting File 2: advs75502‐sup‐0002‐FigureS1.docx. [file ADVS-13-e75502-s001.docx]

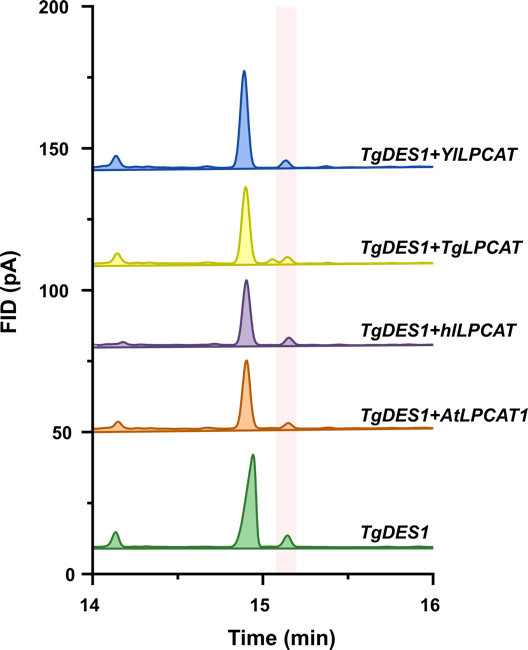


Fig. S1. Testing of LPCAT of different sources. The horizontal axis represents the peak time, and the vertical axis represents the FID response value.


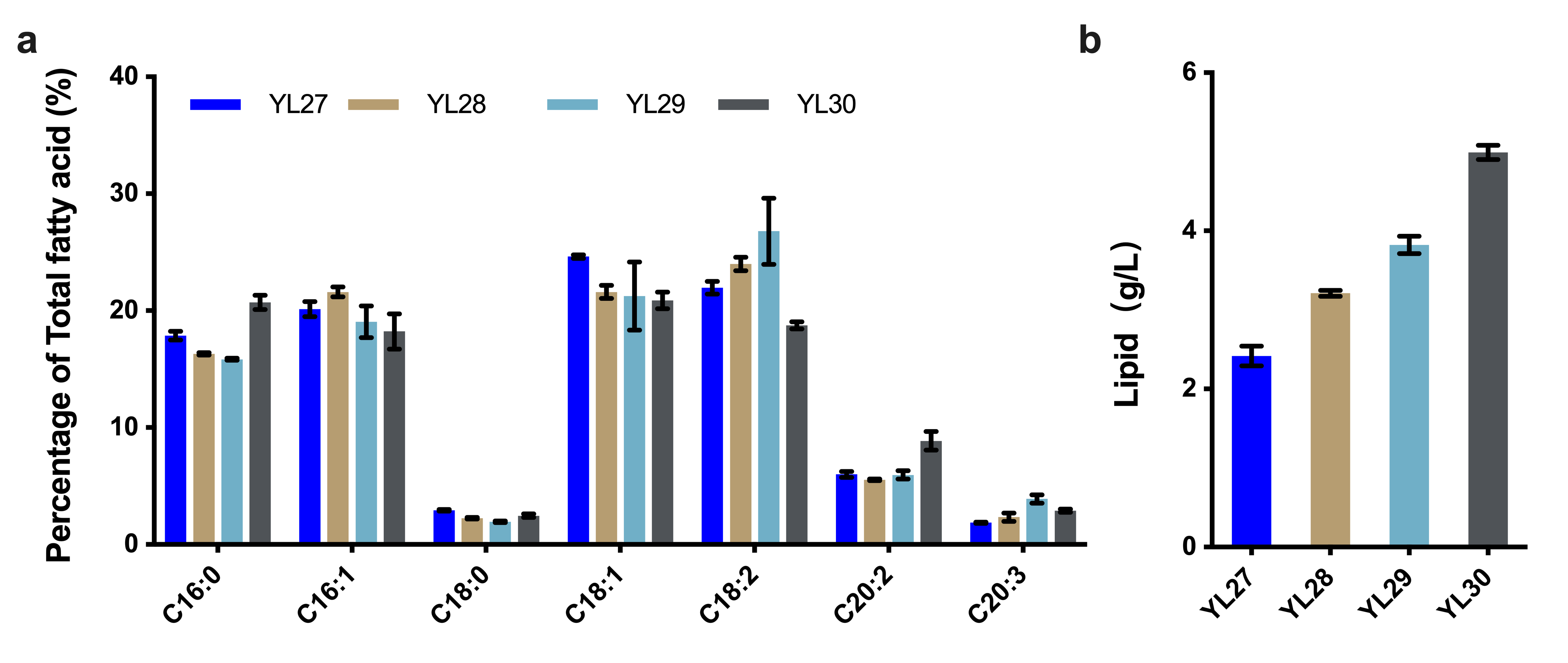


**Fig. S2. The fatty acid composition and lipid titer in engineered strains in YPD medium.** All data are represented as the means of n = 3 biologically independent samples and error bars show the standard deviations.


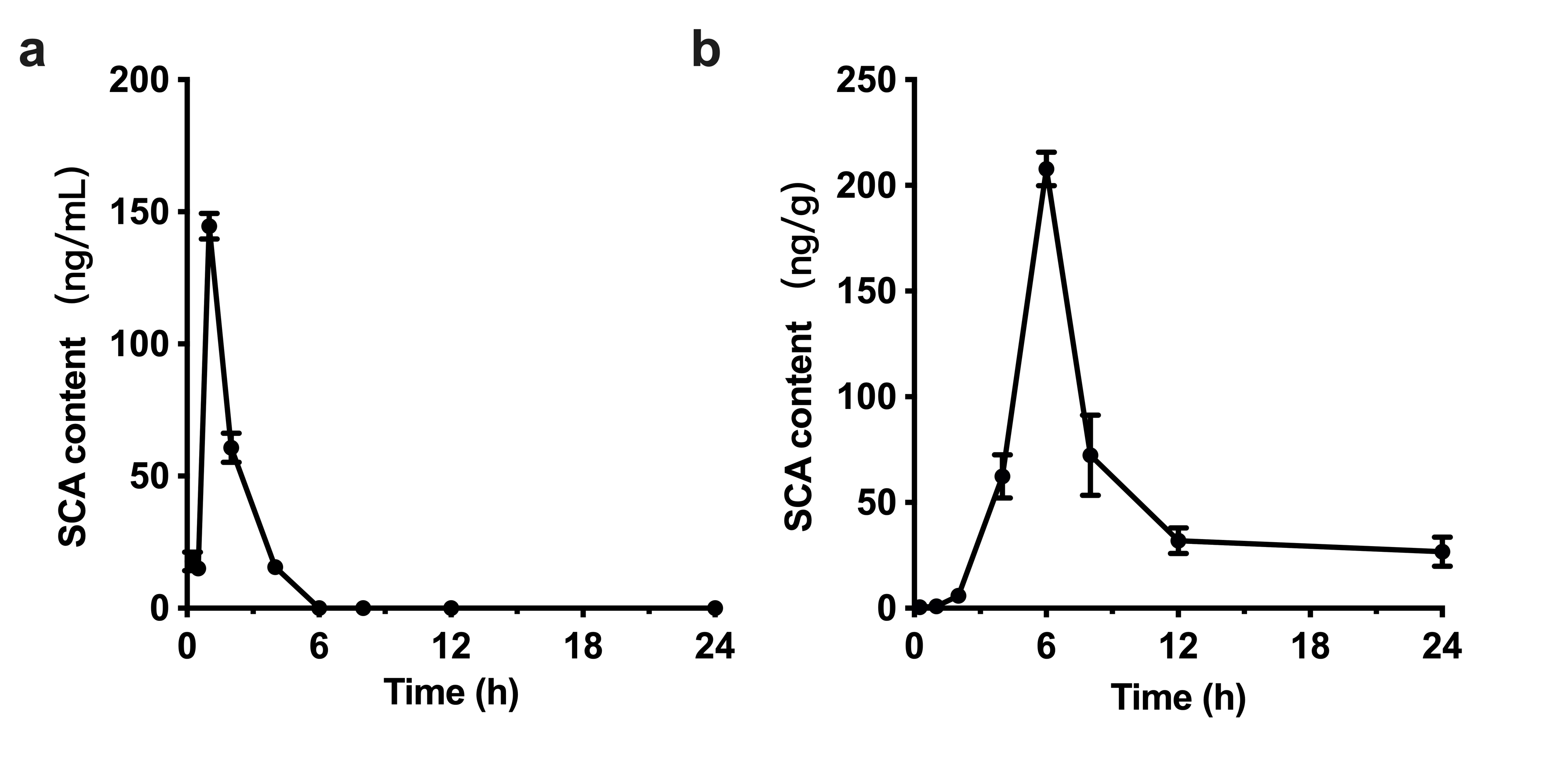


**Fig. S3. Drug-time curves after methyl SCA administration in mice.** a, plasma and b, colon. All data are represented as the means of n = 3 biologically independent samples.


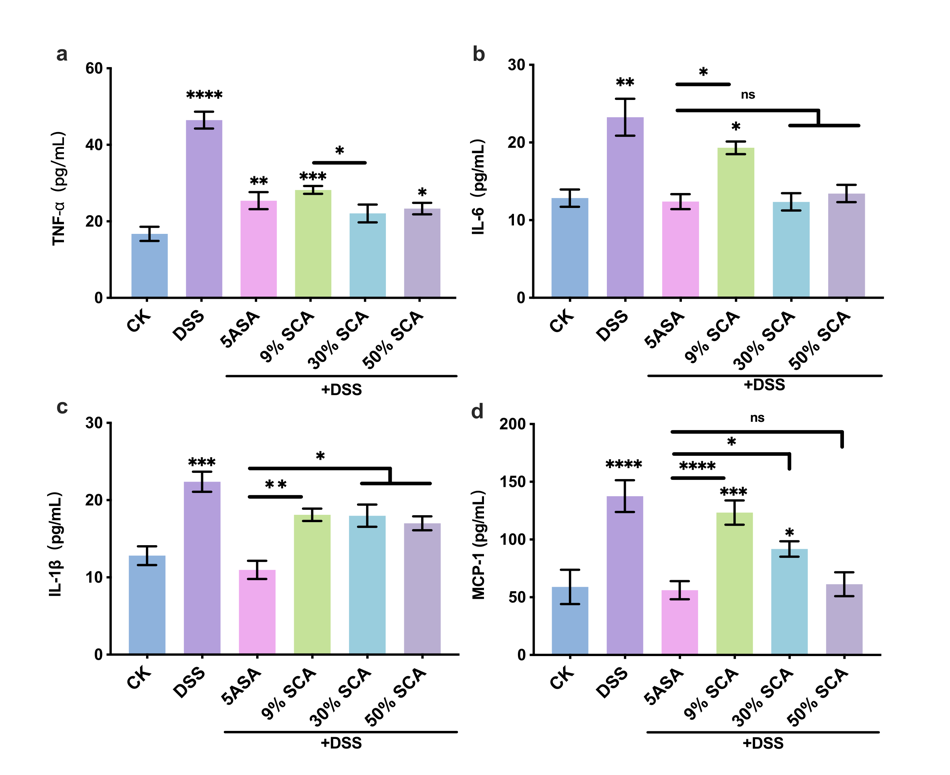


**Fig. S4. Serum concentrations of pro-inflammatory cytokines in DSS-induced colitis mice treated with increasing doses of sciadonic acid (SCA).** a, TNF-α; b, IL-6; c, IL-1β; d, MCP-1. All data are represented as the means of n = 3 biologically independent samples. Data represents mean ± SD; **p<0.01, ***p<0.001 vs control group (one-way ANOVA).


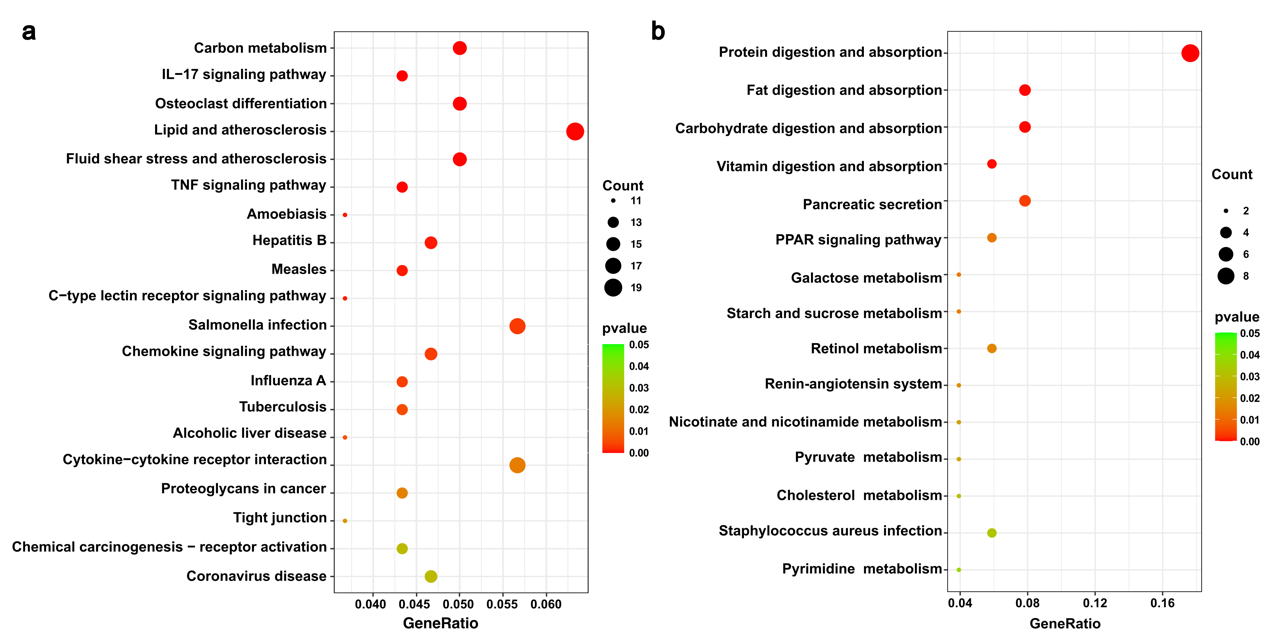


Fig. S5. KEGG pathway enrichment analysis comparing DSS-treated versus control mice and 50% SCA-treated versus DSS-treated mice. Bubble size corresponds to the number of differentially expressed genes (DEGs); color intensity represents the significance level of enrichment (p-value).


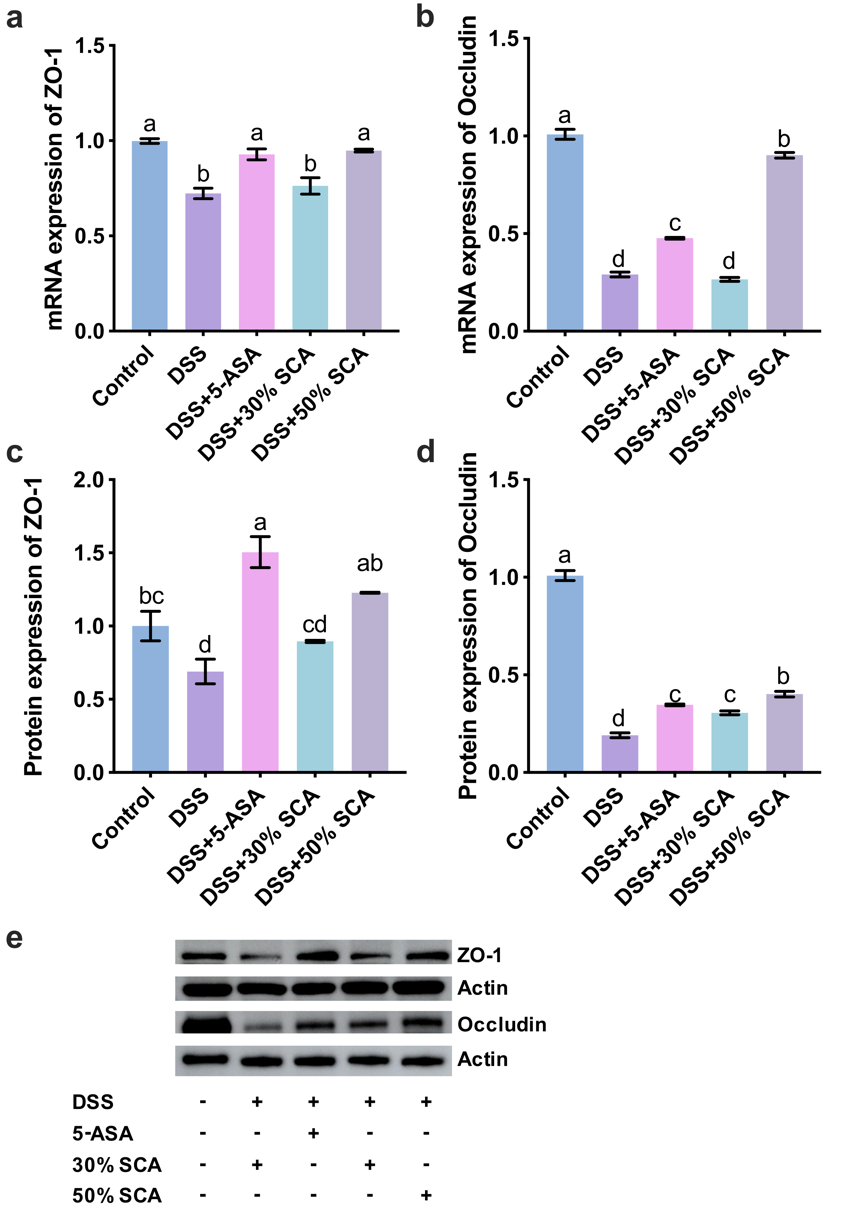


**Fig. S6. Effects of DSS and SCA treatments on ZO-1 and Occludin expression in colonic tissues.** All data are represented as the means of n = 3 biologically independent samples. Different letters indicate a significant difference determined by one-way analysis of variance (ANOVA) at P < 0.05.
